# LPV Control for the Full Region Operation of a Wind Turbine Integrated with Synchronous Generator

**DOI:** 10.1155/2015/638120

**Published:** 2015-03-22

**Authors:** Guoyan Cao, Karolos M. Grigoriadis, Yaw D. Nyanteh

**Affiliations:** Mechanical Engineering in Cullen College of Engineering, University of Houston, N207 Engineering Building 1, 4800 Calhoun Road, Houston, TX 77204-4006, USA

## Abstract

Wind turbine conversion systems require feedback control to achieve reliable wind turbine operation and stable current supply. A robust linear parameter varying (LPV) controller is proposed to reduce the structural loads and improve the power extraction of a horizontal axis wind turbine operating in both the partial load and the full load regions. The LPV model is derived from the wind turbine state space models extracted by FAST (fatigue, aerodynamics, structural, and turbulence) code linearization at different operating points. In order to assure a smooth transition between the two regions, appropriate frequency-dependent varying scaling parametric weighting functions are designed in the LPV control structure. The solution of a set of linear matrix inequalities (LMIs) leads to the LPV controller. A synchronous generator model is connected with the closed LPV control loop for examining the electrical subsystem performance obtained by an inner speed control loop. Simulation results of a 1.5 MW horizontal axis wind turbine model on the FAST platform illustrates the benefit of the LPV control and demonstrates the advantages of this proposed LPV controller, when compared with a traditional gain scheduling PI control and prior LPV control configurations. Enhanced structural load mitigation, improved power extraction, and good current performance were obtained from the proposed LPV control.

## 1. Introduction

Due to multiregional operational characteristics, aeroelastic interactions, and structural flexibility, horizontal axis wind turbines (HAWTs) are modeled as nonlinear and time-varying dynamic systems. Previous work such as in [1–5] examined robust control and gain scheduling control of wind turbines in a single operating region, that is, the partial load or the full load region. Advances in gain scheduling wind turbine control have been achieved in [6, 7] by updating parameters of the controllers according to prespecified functions of measured variable, that is, the wind speed or the rotor speed, instead of interpolating a set of local controllers. However, the corresponding designs were based on very simple wind turbine models. As pointed out in [8], a systematic way of designing such parameter-dependent controllers is within the framework of linear parameter varying (LPV) systems. LPV control is specifically suitable for such plants whose dynamics changes with parameter variation, and the controller is scheduled (adapted) based on measurement of the varying parameters. LPV control is based on systematic design principles and was demonstrated to be effective in such nonlinear and time-varying systems [9–12]. However, past research has focused on a single operating region of the wind turbine, and little effort was spent on finding an active control scheme that can cover the full operating region. In [8], the authors designed an LPV controller for the full operating region of a wind turbine where the LPV control was deduced from the solution of a set of infinite-dimensional LMIs that was reduced to finite-dimensional LMIs by gridding the varying parameter space. In addition, the authors used a set of weighting functions to differentiate three operating conditions: partial load and variable-speed operation, partial load and rated speed operation, and full load operation. However, the LPV control cannot systematically generate a control input for the different operation regions, and the generator torque and pitch angle are varying at the same time in each operating region, resulting in failure to capture the optimal wind power in the partial load region. Additionally, the generator is sometimes operating at a hyperrated condition in the full load operating region. Thus, the LPV control is not fully successful to accomplish the objectives of full region operation. In [11, 12], the authors proposed a control law, which uses a switch logic between two controllers for the partial and the full load regions, to fulfill the control over both regions. However, switching controllers could lead to unsmooth transition and could result in “spike effects.”    For example, large control effort jumps can occur which could induce large forces or momentums at the transition points. In [13, 14], an LPV antiwindup controller was designed to improve the transition operation between the two regions with a two-controller topology. However, the control topology increases the complexity of the control design and brings the issue of the appropriate combination of the controllers.

Additionally, there has been little research effort on the analysis of the electrical system performance when the mechanical subsystem of the wind turbine is controlled. In [15] the authors describe a systematic way of implementing an *H*_*∞*_ control design for a 2 MW double fed inducting generator (DFIG) wind turbine conversion system. However, the authors focused on the electrical performance rather than the effect of the mechanical control on the power generation. In  [16, 17] the authors performed analysis on power extraction and synchronous machine performance, but the analysis was based on low degree of freedom wind turbine models and was without any consideration of load mitigation.

In the present paper, a single LPV controller is designed to meet the operational objectives on the different operation regions. In our design, the LPV model is derived from the state space models extracted by FAST. The FAST (fatigue, aerodynamics, structures, and turbulence) code is a detailed comprehensive high-fidelity wind turbine simulator that is considered the industry standard for simulation of wind turbine systems [18]. The scheduled parameters in the LPV model were regressed as nonlinear functions of the measured wind speeds. Frequency-dependent varying parametric weighting functions were designed to assure smooth transition between the two regions. The LPV controller is constructed based on the solutions of a set of linear matrix inequalities (LMIs) and updated by the prespecified nonlinear functions and the transitional characteristics between the two regions. A synchronous generator model was also considered and integrated to examine the electrical performance corresponding to the generator torque and speed from the LPV control. Simulation of the LPV control is implemented for a 1.5 MW wind turbine model using the FAST code to demonstrate the benefit of the proposed design.

## 2. Model Description and LPV Model

A model-based approach will be considered for the proposed LPV control design. Lumped-parameter models of wind turbine aerodynamic, structural, and vibrating components have been examined in [11, 12, 14, 19]. The following is a brief review of the aerodynamics and generator models used in the present design.

### 2.1. Aerodynamics Model

**Figure 1:**
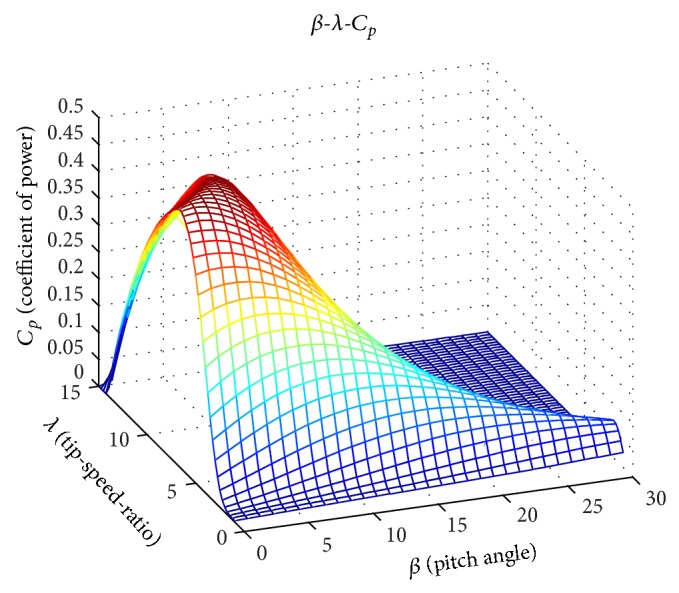
Power coefficient *C*_*p*_ with respect to tip-speed-ratio and pitch angle [20].

The following equations provide the aerodynamic torque Γ_*r*_ on the rotor and the power production *P*_*wt*_, respectively:

(1) $$\begin{array}{c} \Gamma_{r}=\frac{1}{2}\pi r^{3}\rho v^{2}C_{\Gamma }\left(\lambda ,\beta \right), \end{array}$$

(2) $$\begin{array}{c} P_{wt}=\frac{1}{2}\pi r^{2}\rho v^{3}C_{p}\left(\lambda ,\beta \right), \end{array}$$

where *ρ* is the air density, *r* is the radius of the rotor, and *v* is the wind speed. The torque coefficient *C*_Γ_ is given by *C*_Γ_ = Γ_*r*_/(1/2)*πr* ^3^ *ρv* ^2^. The power coefficient *C*_*p*_(*λ*, *β*) is given by *C*_*p*_ = *P*_*wt*_/(1/2)*πr* ^3^ *ρv* ^3^. Let *λ* denote the tips-speed-ratio defined by *λ* = *rω*_*r*_/*v*, where *ω*_*r*_ is the rotor rotation speed and *β* is the blade pitch angle. Then, it follows that *C*_*p*_ = *λC*_Γ_.  Figure 1 shows the power coefficient corresponding to the NREL 1.5 MW baseline wind turbine [20].

### 2.2. Synchronous Generator Model

**Figure 2:**
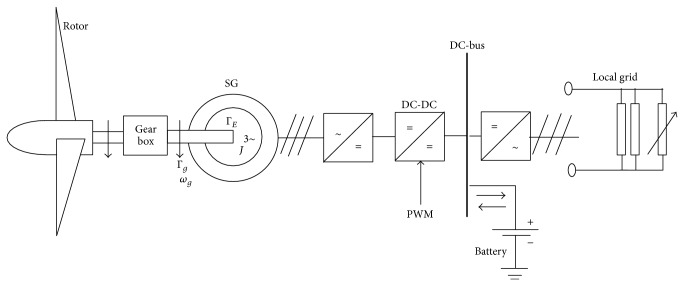
Wind energy conversion system.

The wind energy conversion system with the interconnection of the mechanical components of the wind turbine, generator, and electrical subsystems is shown in Figure 2. The conservation of angular momentum of the generator is presented as in

(3) $$\begin{array}{c} \Gamma_{E}=J\left(\frac{2}{p}\right)\frac{d\omega_{g}}{dt}+\Gamma_{g}, \end{array}$$

where Γ_*E*_ is the electrical torque, *J* is the moment of inertia of the generator, *p* is the number of poles of the generator, *ω*_*g*_ is the generator rotor rotation speed, and Γ_*g*_ is the mechanical torque.

In general, the electrical and electromechanical behavior of most synchronous machines can be predicted from the equations that describe the 3-phase salient-pole synchronous machine [21]. The voltage of the stator windings of a synchronous machine can be expressed in the quadrature-direct (*q* − *d*) axis reference frame as

(4) $$\begin{array}{c} {\overset{-}{V}}_{qdos}=-{\overset{-}{r}}_{s}{\overset{-}{i}}_{qdos}+\omega_{g}{\overset{-}{\lambda }}_{dqos}+\frac{d}{dt}\left({\overset{-}{\lambda }}_{qdos}\right), \end{array}$$

where ${\overset{-}{V}}_{qdos}$, ${\left({\overset{-}{\lambda }}_{dqs}\right)}^{T}=\left[\begin{array}{ccc} \lambda_{ds} & -\lambda_{qs} & 0 \end{array}\right]$, and ${\overset{-}{i}}_{qdos}$ are voltage, flux leakages, and current in the quadrature (*q*), direct (*d*), and original directions (*o*), respectively, in the stator (*s*) reference frame. Also, ${\overset{-}{r}}_{s}$ is the appropriate turns ratio. The rotor circuit is first referred to as the stator circuit and then the rotor voltage equations are expressed in the rotor reference frame with the current runs-ratio with raised index *r* to denote the rotor reference frame and apostrophe to denote the referred quantities in equation as

(5) $$\begin{array}{c} {\overset{-}{V}}_{qdr}^{\prime r}={\overset{-}{r}}_{r}^{\prime }{\overset{-}{i}}_{qdr}^{\prime r}+\frac{d}{dt}\left({\overset{-}{\lambda }}_{qdr}^{\prime r}\right). \end{array}$$

For more details, see [21].

### 2.3. Linear Parameter Varying (LPV) Model

FAST linearization is applied in this work to develop the LPV wind turbine model. The FAST code has the capability of extracting linearized state space representations of the complete nonlinear aeroelastic wind turbine by means of perturbing the states variables and control inputs about their respective operating points. Operating points considered in the present work correspond to steady wind speed input in the range of 4–25 m/s.

The number of states in the state space model is determined by the degrees of freedom (DOFs) enabled in the wind turbine model. In this work, 6 DOFs are considered. They are 1 DOF of the top motion for the longitudinal vibration mode of the tower, *y*_*t*_; 2 DOFs of the drivetrain system (the generator rotation, *θ*_*g*_, and the compliant torsional displacement, *θ*_*s*_); and 3 DOFs of the flapwise tip motion for the first vibration mode of the blades, *ξ*_*b*1_, *ξ*_*b*2_, and *ξ*_*b*3_. Therefore, the state vector is

(6) $$\begin{array}{c} X={\left[y_{t},\theta_{g},\theta_{s},\xi_{b1},\xi_{b2},\xi_{b3},{\dot{y}}_{t},\omega_{g/r},\omega_{r},{\dot{\xi }}_{b1},{\dot{\xi }}_{b2},{\dot{\xi }}_{b3}\right]}^{T}. \end{array}$$

The control input is *U*, the generator torque Γ_*g*_, and the collective blade pitch angle *β*, in the partial load region and the full load region, respectively. The disturbance input is the wind speed disturbance *W*. Along with the linearized equations of motion, FAST also generates a linearized system of outputs *Z*. The outputs selected in the present wind turbine model are the nonrotational tower-top bearing pitch moment, *M*_*t*_, the rotor torque, Γ_*r*_, the rotor speed, *ω*_*r*_, and the blade flapwise moment at the blade root, *M*_*b*_. That is, *Z* = [*ω*_*r*_, *M*_*t*_, Γ_*r*_, *M*_*b*_]^*T*^.

Thus, at an operating point with a steady wind speed, $\overset{-}{v}$, in the range 4–25 m/s, the linearized state space model is

(7) ΔX˙=A−v−ΔX+B−v−1ΔW+B−v−2ΔU,ΔZ=C−v−1ΔX+D−v−11ΔW+D−v−12ΔU.

Applying uniform linearization at the operating points with steady wind speed values spaced every one meter per second from 4 m/s to 25 m/s, 22 sets of state space matrices ($\overset{-}{A}$, $\overset{-}{B}$, $\overset{-}{C}$, and $\overset{-}{D}$) are obtained.

In the state space formulation, we notice that some elements in the state space matrices are nearly constant and some elements are significantly varying with wind speed. Thus, the LPV model can be formalized by means of keeping the constant elements and replacing the varying elements by nonlinear regression functions. The argument of the regression functions is the steady wind speed. The varying elements are then considered as scheduling parameters.

Therefore, the corresponding LPV model is

(8) $$\begin{array}{c} P\left(\tilde{v}\right)=\left\{\begin{array}{cc} \dot{x}=A\left(\tilde{v}\right)x+B_{1}\left(\tilde{v}\right)w+B_{2}\left(\tilde{v}\right)u & \\ z=C_{1}\left(\tilde{v}\right)x+D_{11}\left(\tilde{v}\right)w+D_{12}\left(\tilde{v}\right)u, & \\ y=C_{2}x+D_{21}w+D_{22}u, & \end{array}\right. \end{array}$$

where $\tilde{v}$ is the wind speed measurement, and

(9) Av~=000000100000000000010000000000001000000000000100000000000010000000000001−6.850−509.90.420.420.42−0.090.150.760.010.010.0100136500000a88v~24.37000−0.060−15640a94v~a95v~a96v~−0.01a98v~−28.0600017.57−1.230103−69.74−2.15−2.15−9.13a108v~71.56a1010v~−0.06−0.0617.57−0.2130103−2.15−2.15−69.74−9.13a118v~71.56−0.06a1111v~−0.0617.56−0.1730103−2.15−69.74−2.15−9.13a128v~71.55−0.06−0.06a1212v~,B1v~=08×1b191(v~)b1101(v~)b1111(v~)b1121(v~),  B2v~=07×107×1−0.0002143b282v~0.0002143b292v~0b2102v~0b2112v~0b2122v~,C1v~=00000009.5499.549000128.010c123(v~)749.47−2.17−2.29−4.4c128(v~)c129(v~)17.25−0.1−0.10056000000000010000000c141(v~)0−1108.85.55.55.519.81.28−0.48−0.1−0.1−0.1,C2=00000009.5499.549000,D11(v~)=0d1121v~0d1141v~,  D12v~=000d1221v~000d1241v~,D21=0,  D22=00,x=ΔX,  w=v−v~,  u=ΔΓgΔβ.

**Figure 3:**
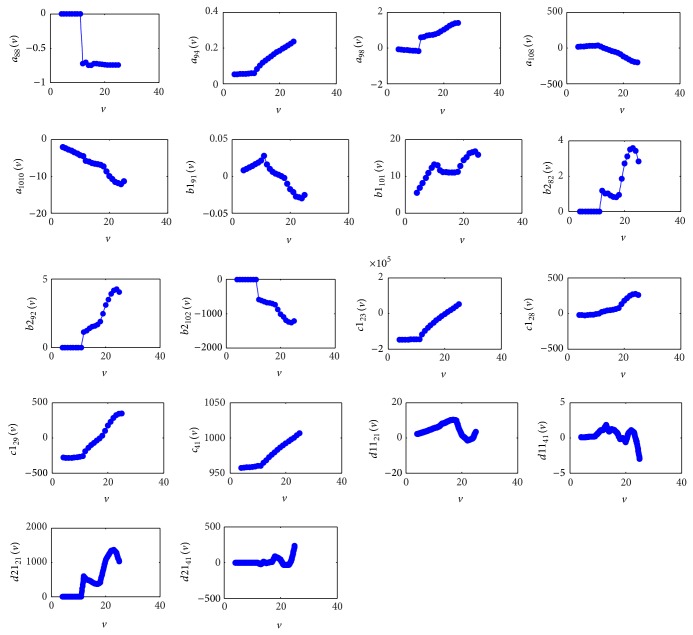
The values of scheduled parameters at the operation points.

In the above LPV model, the scheduled parameters are *a*_88_, *a*_94_ (i.e., equal to *a*_95_ and *a*_96_), *a*_98_, *a*_108_, *a*_1010_ (i.e., equal to *a*_1111_ and *a*_1212_), *b*1_91_, *b*1_101_ (i.e., equal to *b*1_111_ and *b*1_121_), *b*2_82_, *b*2_92_, *b*2_102_ (i.e., equal to *b*2_112_ and *b*2_122_), *c*1_23_, *c*1_28_, *c*1_29_, *c*1_41_, *d*11_21_, *d*11_41_, *d*21_21_  , and *d*21_41_. Their values at every single operating point are shown in Figure 3. The nonlinear regression functions to describe the variability of these elements will be discussed further in Section 3.3.

## 3. LPV Control Design

### 3.1. Control Objectives

The wind turbine control objective can be summarized as achieving the maximum power point tracking (MPPT) in the partial load region and structural load mitigation in the full load region. In order to attain MPPT in the partial load region, the blade pitch angles should be kept at their optimal value, *β* ^*^, and the generator torque is controlled to regulate the rotor speed to track the optimal rotor speed corresponding to the optimal tip-speed-ratio, *λ*_opt_. In the full load region, the blade pitch angle is controlled to mitigate the structural loads, while the generator is kept working at rated speed. Additionally, the operation in both regions should be wind noise insensitive and be safe by limiting the force resulting from sudden wind gusts.

### 3.2. LPV Control Structure

**Figure 4:**
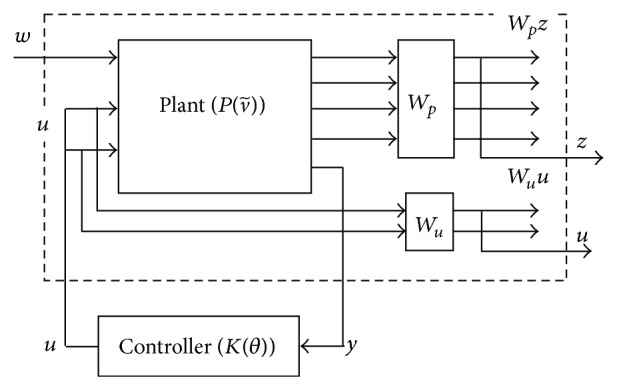
The lower LFT control structure with augmented plant.

In order to meet the control objectives and provide a smooth transition between the two operation regions, frequency-dependent varying scaling parametric weighting functions are designed. Traditional frequency-dependent weighting functions can be used to regulate different frequency components of a signal. Frequency-dependent weighting functions with high-frequency-pass property are used to attenuate high-frequency components of plant output signal in *H*_*∞*_ control [22]. Thus, we design such frequency-dependent weighting functions for the rotor speed, the generator torque, and the blade pitch angle to attenuate the impact of wind variability on the power generation and the control actuators. Taking practical controller implementation into account, the frequency-dependent weighting functions are designed as (*τ*_1_ *s* + 1)/(*τ*_2_ *s* + 1) in the Laplace notation. The weight parameters are chosen as *τ*_1_ = 5 and *τ*_2_ = 0.1. After applying the weighting functions on the plant outputs and the control inputs, the LPV model becomes an augmented model of 14 states. The lower linear fractional transformation (LFT) control structure of the augmented model is presented as in Figure 4.

Noticing that a high-pass frequency weighting function can regulate high-frequency components of a signal, a varying scaling parameter with respect to wind speed can be used in the weighting function to penalize a control input in an operating region. Two varying scaling parameters, *W*_*g*_ and *W*_*β*_, were designed as weighting functions for the generator torque channel and the pitch angle channel, respectively. The design concept is as follows: the magnitude of *W*_*g*_ is selected to be a constant in the partial load region and another constant in the full load region. This is because the effectiveness of the generator torque control is equal when the wind turbine is operated in the same operation region. The constant weight in the partial load region is selected to be less than the constant in the full load region, which is effective to regulate the generator torque to be insensitive in the full load region. With the same logic, the magnitude of *W*_*β*_ is selected to be a constant in the partial load region and another constant in the full load region, but conversely the constant in the full load region is less than the constant in the partial load region. In addition, the scale of the magnitudes of *W*_*g*_ and *W*_*β*_ plays a normalization role to balance the regulation of the generator torque and the blade pitch angle, as the magnitude of the generator torque is very different from the magnitude of the blade pitch angle. In addition, in order to assure a smooth transition between the two operation regions, the two varying scaling parameters, *W*_*g*_ and *W*_*β*_, should be smooth functions of the steady wind speed.

**Figure 5:**
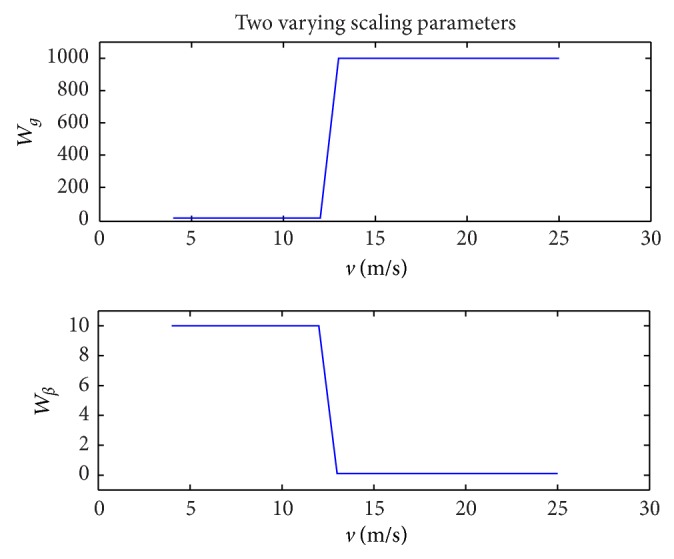
Prescribed functions for varying scaling parameters of weighting functions.

Based on the above considerations, we select the two varying scaling weights, *W*_*g*_ and *W*_*β*_, to be sigmoid functions, since sigmoid functions with large slope are able to provide weight variability in the full region to satisfy the design objectives. The weight selection is as follows: $W_{g}(\tilde{v})=10+\left(1000/\left(1+e^{\left(-10\left(\tilde{v}-12\right)\right)}\right)\right)$ and $W_{\beta }(\tilde{v})=10.1+\left(-10/\left(1+e^{\left(-10\left(\tilde{v}-12\right)\right)}\right)\right)$, shown in Figure 5.

In addition, we replace the first output to be the error of the rotor speed tracking,

(10) $$\begin{array}{c} e_{r}=\left\{\begin{array}{cc} \omega_{r}-\frac{\lambda^{*}}{R}\tilde{v}, & \text{in}\;\text{partial}\;\text{load}\;\text{region}\\ {\omega_{r}-\omega }_{\text{rated}}, & \text{in}\;\text{full}\;\text{load}\;\text{region}, \end{array}\right. \end{array}$$

to better represent the control objective of tracking of the rotor speed. Thus, the augmented output vector is formulated as

(11) Z^=50s+10s+10110011001100Wg50s+10s+10Wβ50s+10s+10,KLkk50s+10s+10110011001100Wg50s+10s+10Wβ50s+10s+10eωrMbTrMtΓgβ.

**Figure 6:**
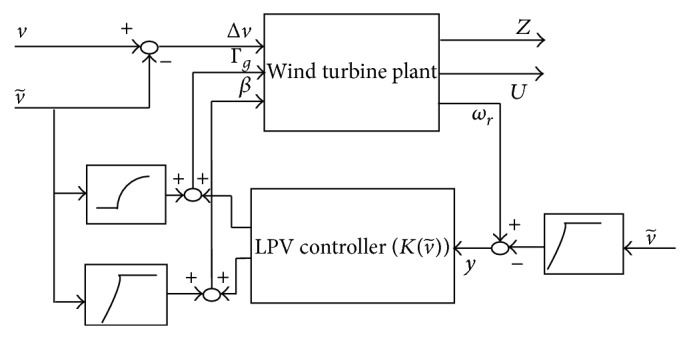
LPV control structure.

Therefore, the LPV control structure has the block diagram shown in Figure 6. where the LPV controller $K\left(\overset{-}{v}\right)$ is

(12) $$\begin{array}{c} K\left(\tilde{v}\right)∶=\left\{\begin{array}{cc} {\dot{x}}_{k}=A_{k}\left(\tilde{v}\right)x+B_{k}\left(\tilde{v}\right)y, & \\ u=C_{k}\left(\tilde{v}\right)x+D_{k}\left(\tilde{v}\right)y. & \end{array}\right. \end{array}$$

### 3.3. LPV Controller Design via LMIs

Based on the following result [23–25], gain scheduled controllers for LPV systems can be obtained by means of solving a linear matrix inequalities (LMIs) problem.

**Theorem 1 .**

Given the open-loop system in (8) and a scalar *γ* > 0, there exists a parameter-dependent controller as in (12) that guarantees closed-loop system stability and *H*_*∞*_-norm less than *γ* if there exist parameter-dependent symmetric matrices *X* and *Y* and parameter-dependent matrices ${\hat{A}}_{K}$, ${\hat{B}}_{K}$, ${\hat{C}}_{K}$, and *D*_*K*_ such that for all pairs of ($\tilde{v}$, $\dot{\tilde{v}}$) in their hypercube space *V* × *V*_*d*_ the following infinite-dimensional LMI problem holds true:

(13) X˙+XA+B^KC2+⋆∗∗∗A^KT+A+B2DKC2−Y˙+AY+B2C^K+⋆∗∗XB1+B^KD21TB1+B2DKD21T−γI∗C1+D12DKC2C1Y+D12C^KD11+D12DKD21−γI<0,  XIIY>0,

where (⋆) denotes the transpose of the nonsymmetric terms followed by it and ∗ is used to denote the entries which make the entire matrix to be symmetric.

The lower and upper limit for all pairs ($\tilde{v}$, $\dot{\tilde{v}}$) are defined as follows:

(14) $$\begin{array}{c} \tilde{v}\left(t\right)\in \left[4\;\text{m}/\text{s},25\;\text{m}/\text{s}\right],\;\forall t\geq 0,\\ \dot{\tilde{v}}\left(t\right)\in \left[-3\;\text{m}/{\text{s}}^{2},3\;\text{m}/{\text{s}}^{2}\right],\;\forall t\geq 0. \end{array}$$

Finally, an LPV controller is readily obtained with the following two-step scheme.

(i) Solve for *N* from the formula *I* − *XY* = *NM* ^*T*^, where *M* = *I*.
(ii) Compute *A*_*K*_, *B*_*K*_, and *C*_*K*_ with
  (15) AK=N−1A^K−XA−B2DKC2Y−B^KC2Y−XB2C^K,BK=N−1B^K−XB2DK,CK=C^K−DKC2Y.

The above LMIs formulation is infinite-dimensional as the LMIs should be solved at all pairs of ($\tilde{v}$, $\dot{\tilde{v}}$) in the defined region (see (14)). In order to reduce the infinite-dimensional LMIs to a finite-dimensional problem, the scheduling variables are selected to “mimic” the parameter dependence of the LPV model in the parameter-dependent matrices ($X,Y,{\hat{A}}_{K},{\hat{B}}_{K},{\hat{C}}_{K}$, and *D*_*K*_) [23]. The parameter-dependent matrices can be rewritten in an affine fashion hereafter:

(16) A^K(v~)∶=A^K,0+∑i=1Nρi(v~)A^K,i;B^K(v~)∶=B^K,0+∑i=1Nρi(v~)B^K,iC^K(v~)∶=C^K,0+∑i=1Nρi(v~)C^K,i;DK(v~)∶=DK,0+∑i=1Nρiv~DK,iklXv~∶=X0+∑i=1Nρiv~Xi;  Y∶=Y0,

where $\rho_{i}\left(\tilde{v}\right),\;\;i=1,\ldots ,N$, are scheduled variables and $\tilde{v}$ is the averaged wind speeds. The wind speed measurement is assumed to be known in this design; however, it is not easily measured directly. Novel techniques have been proposed such as LIDAR (light detection and ranging) systems to measure wind speed [26, 27] in wind turbines. It is noted that, in the proposed LPV control, the controller is scheduled by a 10-second averaged wind speed measurement. Therefore, the above methods would be capable to provide qualified wind speed measurement information.

The overall LPV control design procedure is described as follows.

**Step 1 .**

Define a uniform grid *G* for the value set of $\tilde{v}$ and, at each value of $\tilde{v}$, the extreme points of $\dot{\tilde{v}}$, denoted by *T*, are checked.

**Step 2 .**

Minimize *γ* subject to the LMI constraints associated with *G* × *T*.

**Step 3 .**

Check the constraints at a denser grid. If the new *γ* is shown to have converged to the *γ* from Step 2, then the solution is used to construct the LPV controller.

**Step 4 .**

If Step 3 fails, increase the grid density and return to Step 2.

**Figure 7:**
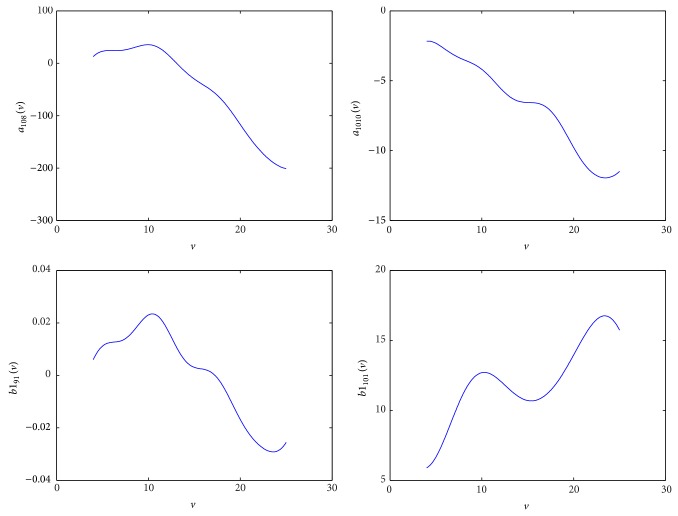
Nonlinear regress functions of the major varying elements.

Before solving the LMIs, the scheduling variables, $\rho_{i}(\tilde{v})$, should be selected for the parameter-dependent matrices (see (16)). It is noted that the scheduling variables selection is based on the scheduled parameters in the LPV model. That is, in principle, the 18 scheduled parameters in the LPV model should be selected as scheduling variables. However, such a larger number of scheduling variables will greatly increase the computation complexity of the design. Thus, we identify the significantly varying scheduled parameters out of all the scheduled parameters and select them as scheduling variables in the LPV controller design. When we are solving the LMIs problem and gridding the space of the time-varying parameter, $\left(\tilde{v}\right)$, all the scheduled parameters in the LPV model ($A_{k}\left(\tilde{v}\right)$, $B_{k}\left(\tilde{v}\right)$, $C_{k}\left(\tilde{v}\right)$, and $D_{k\left(\tilde{v}\right)}$) are updated with the gridding points, and the scheduling variables are used in the construction of *X*, *Y*, ${\hat{A}}_{K}$, ${\hat{B}}_{K}$, ${\hat{C}}_{K}$, and *D*_*K*_. Based on the analytical model derived in [11, 19], the significantly varying scheduled parameters are *a*_108_, *a*_1010_ (*a*_1111_, *a*_1212_), *b*1_91_, and *b*1_101_(*b*1_111_, *b*1_121_). After this identification, the number of scheduling variables is reduced to four, but makes the LPV control a little more conservative.  Figure 7 shows the nonlinear regression functions of the major scheduled parameters. The nonlinear regression functions are obtained by Fourier series regression with the coefficients of determination (*R* ^2^), that is, the indicator of goodness of fit [28], greater than 0.98 for each of the regressions. The other scheduled parameters are merely updated in the LPV model at the grid points spaced uniformly at every unit of wind speed during the solution of the LMIs problem.

Considering the usage of the varying scaling parameters in the weighting functions, the scheduling variables are then selected as $\rho_{1}\left(\tilde{v}\right)=a_{108}\left(\tilde{v}\right)$, ${\rho_{2}\left(\tilde{v}\right)=a}_{1010}\left(\tilde{v}\right)$, ${\rho_{3}\left(\tilde{v}\right)=b1}_{91}\left(\tilde{v}\right)$, $\rho_{4}\left(\tilde{v}\right)={b1}_{101}\left(\tilde{v}\right)$, and ${\rho_{5}\left(\tilde{v}\right)=W}_{\beta }\left(\tilde{v}\right)$. The LMIs design problem (see (13)) is solved with LMI Lab in MATLAB [29].

### 3.4. Closed-Loop Simulation

**Figure 8:**
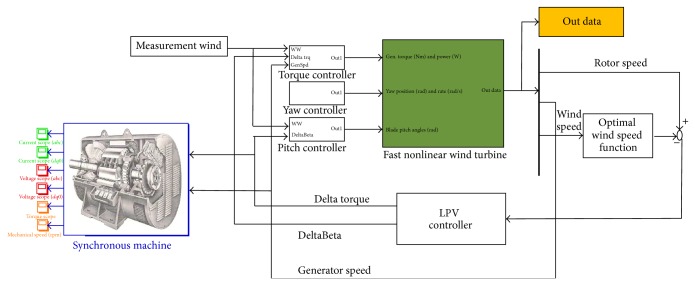
Closed-loop simulation of LPV control in Simulink.

**Figure 9:**
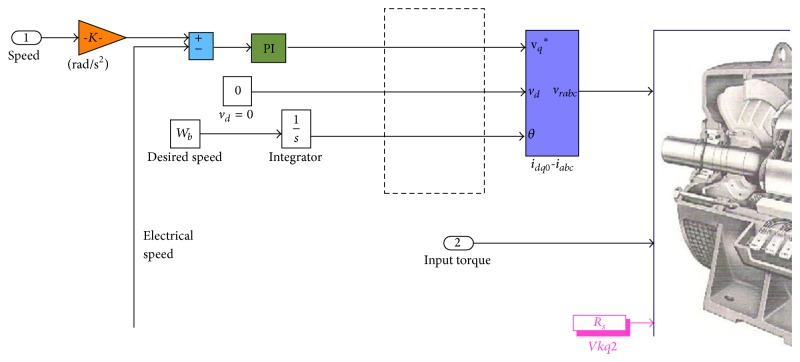
Inner loop of speed control for synchronous generator operation.

To validate the LPV design with a detailed nonlinear model, the closed-loop simulation is implemented in MATLAB/Simulink and coupled with the FAST code as shown in Figure 8. A synchronous generator model is connected to the closed-loop system in order to examine the corresponding electrical performance to the generator torque and the generator speed. The synchronous generator model is constructed based on the mathematical model described in (3)–(5). The given generator speed from the LPV control defines a reference for the generator operation with the mechanical torque equal to the given generator torque. To simplify the discussion, during operation, an assumption may be made that there is unlimited current available through a load commutated AC drive. A PI control loop is designed to determine the current command to achieve a desired generator speed profile. The PI control loop output can, therefore, directly represent the *q*-axis current command of the machine as shown in Figure 9. The current command is translated into a voltage output from the drive that drives the speed dynamics of the machine.

## 4. Simulation Results

In this section, simulation results of the proposed LPV control design are presented to validate the design. In order to show the advantages of the proposed LPV control, comparisons with an LPV control proposed in [8] and a gain scheduling PI control presented in [30, 31] are discussed. It is noted that the proposed LPV control formulation, solution, and validation differ significantly from the approach in [8]. The LPV model in [8] was deduced from the lump model interconnections of the subsystems of a wind turbine. The wind speed assumed available online was used to schedule the LPV model and the LPV controller. Closed-loop simulation was implemented on lumped parameter model that was used for design. Finally, the LPV controller was deduced from a two-stage LMIs formulation. Then, based on the solution of the first stage, a second stage of LMI was formed for solving the controller variables. The two-stage LMIs were iteratively implemented to minimize the *γ* value and finally solve the controller variables. Gridding on the time-varying parameter space was used, and convexity between any two neighbor gridding points was necessary to reduce the infinite-dimensional LMIs to finite dimension. In our design, the LPV model is constructed from linearized state space models extracted by the FAST code and the parameters for LPV design are selected to be varying elements of the corresponding state space matrices. The state matrices of the LPV controller and the Lyapunov function variables are solved directly from the LMIs formulation once the solution is feasible. Closed-loop simulation to validate the designs was implemented on the high-fidelity FAST wind turbine simulation. In addition, both designs use weighting functions to penalize high-frequency elements of the plant outputs. However, in our design, the frequency-dependent varying scaling parametric functions are not only expanded with respect to the scheduling variables, but also designed for choosing the control input, for the respective operation region. Finally, the proposed formulation considers explicitly the interconnection with a synchronous generator to examine the electrical subsystem performance.

### 4.1. Gain Scheduling PI Control

**Figure 10:**
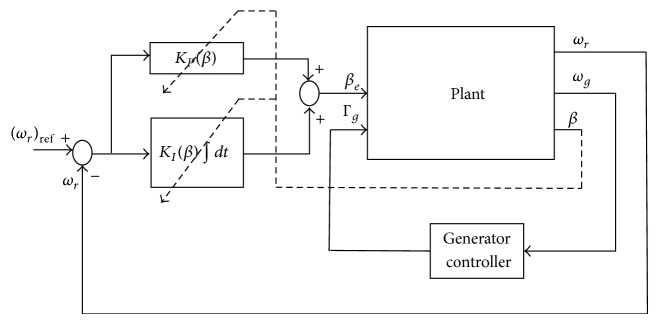
Implementation structure for gain scheduling PI control.

A gain scheduled PI controller composed of a generator torque controller and a gain scheduling PI pitch angle controller (Figure 10) is considered [30]. It is characterized as

(17) $$\begin{array}{c} \Gamma_{g}=\left\{\begin{array}{cc} a*{\left(\omega_{g}\right)}^{2}, & \text{if}\;\omega_{g}<{{(\omega }_{g})}_{\text{rated}}\\ {{(\Gamma }_{g})}_{\text{rated}}, & \text{if}\;\omega_{g}\geq {{(\omega }_{g})}_{\text{rated}} \end{array}\right.\\ \Delta \beta =K_{P}\left(\beta \right)\Delta \omega_{r}+K_{I}\left(\beta \right)\overset{t}{\underset{0}{\int }}\Delta \omega_{r}dt, \end{array}$$

where *a* = (Γ_*g*_)_rated_/((*ω*_*g*_)_rated_)^2^, *K*_*P*_(*β*) = *k*_1_/−(∂*P*/∂*β*)(*β*), and *K*_*I*_(*β*) = *k*_2_/−(∂*P*/∂*β*)(*β*). The term (∂*P*/∂*β*)(*β*) is the sensitivity of the aerodynamic power to rotor-collective blade pitch angles, where *k*_1_ and *k*_2_ can be chosen according to the criteria in [30, 31]. The terms *k*_1_ and *k*_2_ are defined as

(18) k1=2IDrivetrainωratedξφωφNGear,k2=IDrivetrainωratedωφ2NGear,

where *ξ*_*φ*_ and *ω*_*φn*_ are the damping ratio and natural frequency of the lumped second order drivetrain system, respectively; *φ* is the displacement of the drivetrain rotational flexibility, *N*_Gear_ is the gear box ratio, and *I*_Drivetrain_ is the drivetrain inertia relative to the high-speed shaft.

### 4.2. Closed-Loop Simulation Results

The test wind turbine model selected is the NREL 1.5 MW WindPACT and its major properties are provided in Table 1.  Table 2 shows the synchronous generator parameters.

In order to make a clear comparison between the LPV control in [8] and the proposed LPV control in this work, we use LPV1 to denote the LPV controller in [8] and LPV2 for the LPV control in this work. In the following comparisons, solid blue line, the dash green line, and the dash dotted red line represent the result from the gain scheduling PI control, LPV1, and LPV2 control, respectively.

**Figure 11:**
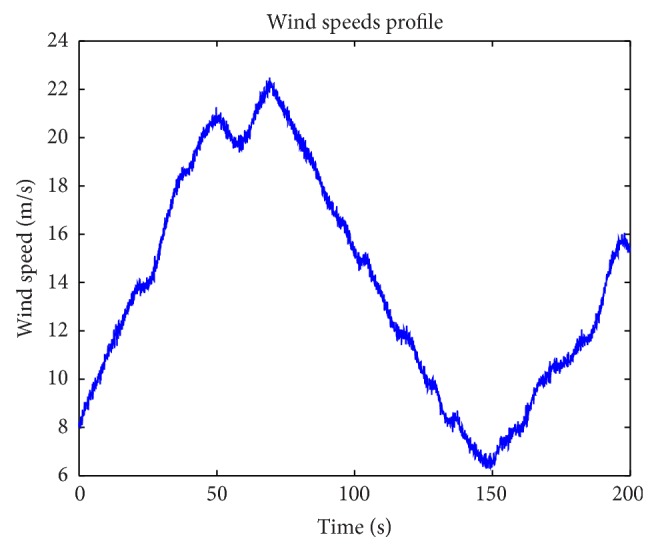
Tested wind profile.

**Figure 12:**
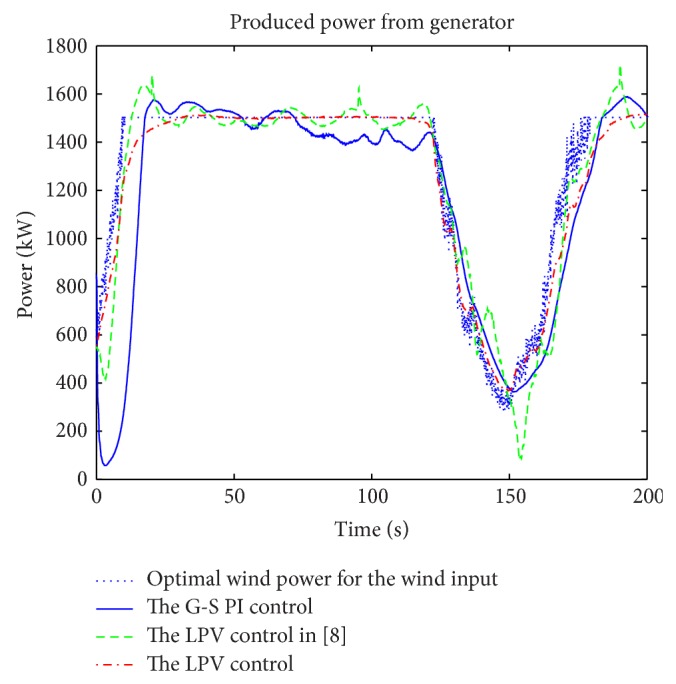
Simulation results of the produced power.

**Figure 13:**
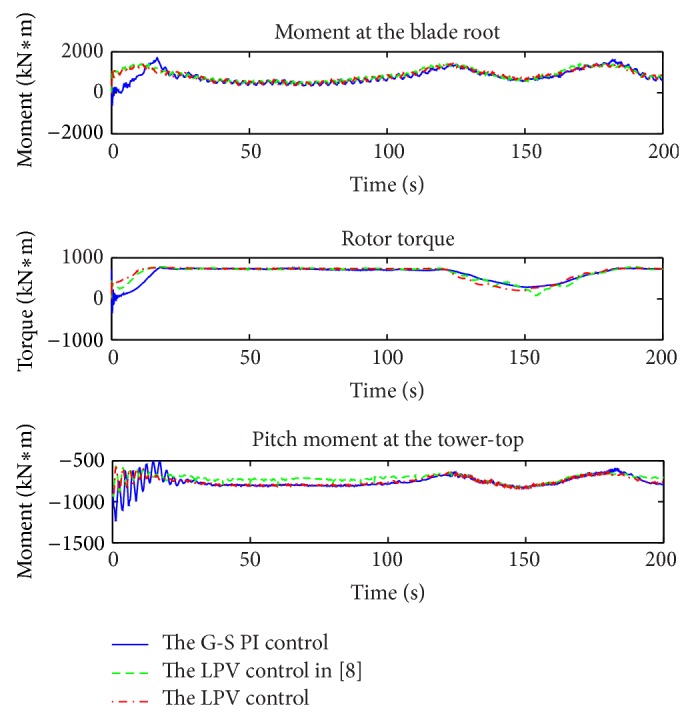
Simulation results of outputs.

**Figure 14:**
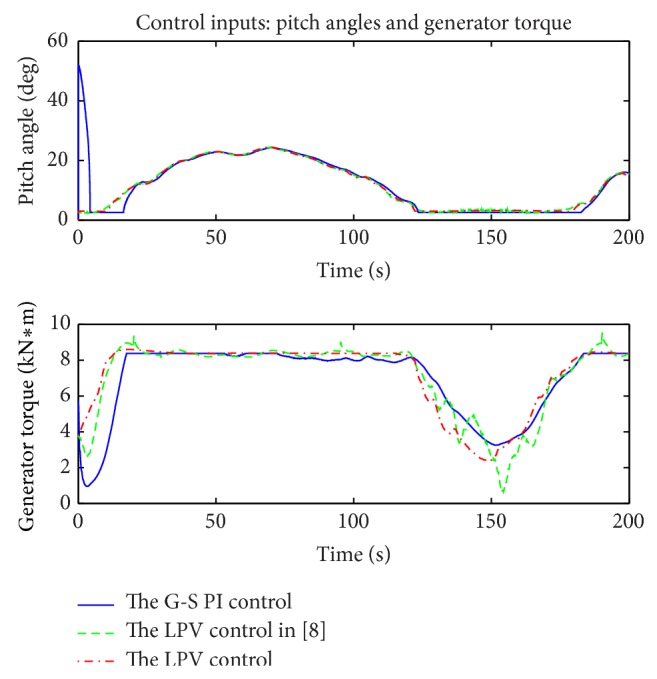
Simulation results of generator torque and pitch angles.

**Figure 15:**
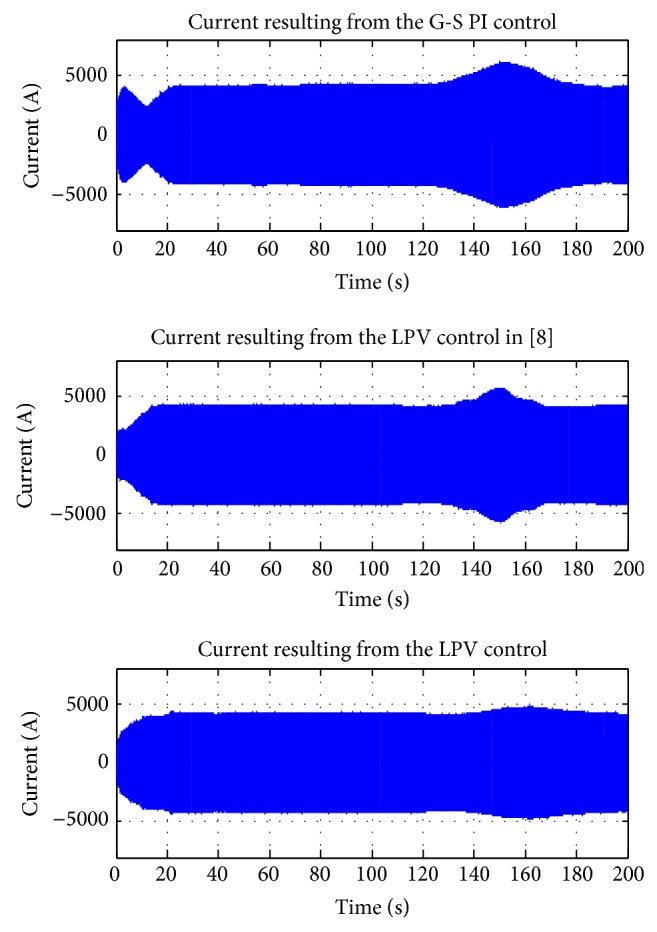
Simulation results of currents.

A wind profile, covering both the partial and the full load regions as in Figure 11, was used for the simulations. The simulation results of power extractions, control inputs, and plant outputs are shown in Figures 12, 13, and 14, respectively. The corresponding electrical subsystem performance is shown in Figure 15.

In Figure 12, the dotted blue line represents the optimal wind power for the given wind speeds. The power extraction from LPV2 is almost equal to the optimal wind power, except for the high-frequency parts of the optimal wind power. However, the “rising time” from LPV2 is a bit longer than the optimal values, and it is significantly better than that from the gain scheduling PI control. The average power extraction from LPV1 is larger than that from LPV2; additionally, LPV1 does not track the optimal wind power as well as LPV2 resulting in the generator sometimes working at hyperrated condition in the full load operation region; see Figure 14.

In Figure 13, the simulation results of the loads, including the moments at the blade root, rotor torque, and pitch moment, are compared. The magnitudes of the loads from LPV1 and LPV2 vary in narrower regions than the corresponding magnitudes from the gain scheduling PI control. Another observation is that the “spike” effects of the gain scheduling PI control are obvious at the transition point of two regions; however, LPV2 smoothly addresses the transitions. It is noticed that the magnitude of the pitch moment at tower-top of LPV1 is smaller than LPV2, but the “spike” effect is significantly larger at the transition point of the partial load and rated speed operation and the full load operation.

Control inputs including the generator torque and the pitch angles are presented in Figure 14. In the gain scheduling PI control, the proportional term *K*_*P*_ and integral term *K*_*I*_ are dependent on the sensitivity of the power to pitch angles ∂*P*/∂*β*. The result shows that the gain scheduling PI control is sensitive to the initial value of the pitch angles. The LPV control, on the other hand, is insensitive to the initial conditions of the pitch angles as demonstrated by the results. When comparing the generator torques, for the gain scheduling PI control, the generator speed is confined by the pitch angles due to the impact of the pitch angles on the aerodynamic torque. When pitch angle control is inappropriate, the generator speed is affected, which causes the generator torque controller fail to track the optimal power, as demonstrated by the results. Oppositely, for the LPV control, the generator torque and pitch angle are independently considered, and, therefore, LPV2 provides two degrees of freedom control to simultaneously realize multiple performance objectives. In LPV1, it is noticed that the pitch angle is at the same time varying in the partial load region which is avoided in LPV2; in LPV1, the generator torque is also varying and even sometimes working over rated condition in the full load region, but in LPV2 it is perfectly denied.

Table 3 summarizes the comparison results of the gain scheduling PI control and LPV1 over LPV2. The tower-top pitch moment energy of LPV1, in *L*_2_ norm sense, is better than that of LPV2. At nearly the same pitch angle condition, the tower-top pitch moment should be nearly the same, but when generator absorbs the load and transforms the load into generator torque, the performed load in LPV1 greatly reduced. However, when the load is compared in *L*_inf⁡_ norm sense, which focuses more on peak energy, they are nearly the same.

Figure 15 provides the simulation results of the currents produced by the synchronous generator connecting the gain scheduling PI control loop and the LPV control loop. It can be seen that the current of the generator connecting the LPV2 loop performs better than the gain scheduling PI control loop and the LPV1 controller. The generator connected with the LPV2 control produces current which rises smoothly to the required value and remains stable despite the wind power variation; additionally, the corresponding rising rate and magnitude are much smaller than the gain scheduling PI control and the LPV1 control benefiting the generator operation and ease of the current converter tuning.

**Figure 16:**
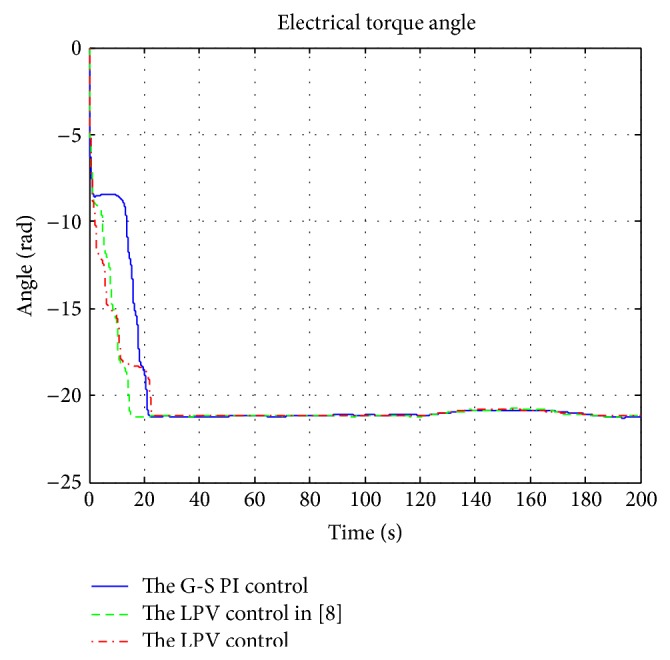
Simulation results of electrical torque angle.

Figure 16 provides the electrical torque angle of the generator under the three difference control schemes. The torque angle is the angle difference, in radians, between the rotor electrical angular displacement and the angular displacement of the terminal voltage of the generator. At steady state, the torque angle has a constant value. The rising time refers to the time taken by the electrical torque to change from 10% to 90% of its steady state value. The settling time corresponds to the time taken for steady state voltage conditions to be established in the machine. The rising time and settling time are 11 sec and 15 sec for the LPV1 control, 18 sec and 23 sec for the gain scheduling control, and 12 sec and 22 sec for the LPV2 control. In each of the cases shown in the figures, the torque angle undergoes about 3 revolutions before settling down to steady state conditions.

**Figure 17:**
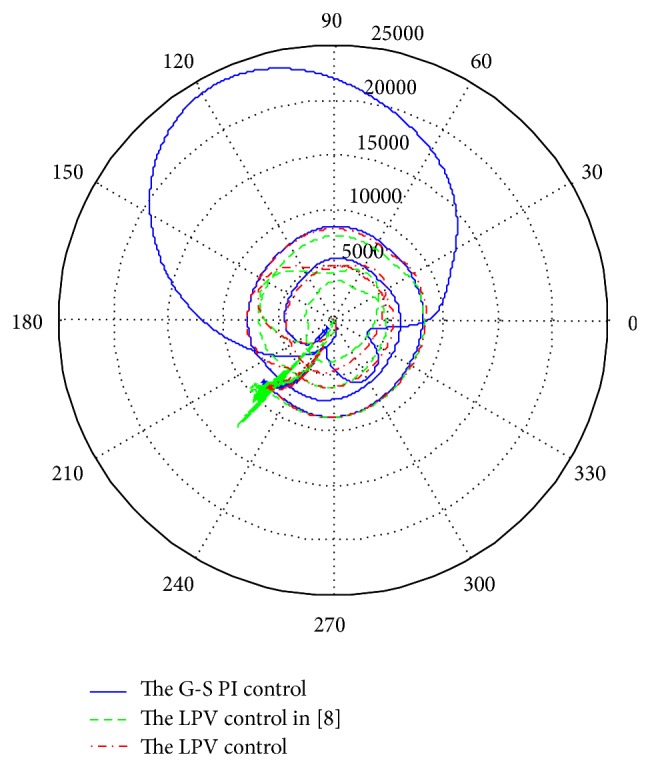
Electrical torque versus torque angle.

Figure 17 provides the comparison of electrical torque with respect to the torque angle of the generator. The polar plot clearly shows the transient responses of the electrical torque under the three control cases. It is desired that the electrical toque performs as smoothly as possible to protect the operation of the generator, and the magnitude of the electrical torque should be as close as possible to the magnitude of the mechanical torque to ensure the generator is working at the determined conditions. From the comparison, we can observe that LPV2 is smoother and its magnitudes are closer than the other two control schemes. Even though the settling time of the LPV1 is smaller than LPV2, the big variation of electrical torque is worse for the operation of the generator.

In conclusion, considering the coupled mechanical load mitigation and the electrical power generation aspects, the proposed LPV2 controller is demonstrated to be more effective than the gain scheduling PI control and the LPV1 control. The multiple objectives of operation in optimal power tracking and loads mitigation are all realized by this LPV control which covers the full operating region.

## 5. Conclusion

A linear parameter varying controller is examined in this work for the full operating region of a wind turbine integrated with synchronous generator. In summary, compared to previous approaches, the proposed LPV control meets multiple performance objectives of optimal power tracking and structural load mitigation. FAST linearization extracts the state space model at every operating point, from which the LPV model is derived. In the developed LPV model, the varying parameters of the state space representation are regressed as nonlinear functions of the measured wind speed and used as scheduling variables. Frequency-dependent varying scaling parametric weighting functions are designed in the formulation of the LPV control to reject the disturbance of high-frequency wind noise and assure smooth transition between the two operation regions. In the high-fidelity FAST simulations of the LPV control, closed-loop performance including the generator current is examined with a synchronous generator model. Simulation of the closed-loop LPV control demonstrates the advantage of the proposed LPV design.

## Figures and Tables

**Table 1:** Principal data of test wind turbine WindPACT 1.5.

WindPact 1.5 model parameter	

Rated electrical power	1500 KW
Rotor diameter	70.0 m
Hub height	84.0 m
Variable rotor speed range	10.0–20.0 rpm (±10%)
Rated rotor speed	20.0 rpm
Tilt angle	5.0 deg
Cone angle	0.0 deg
Blade mass	3912 kg
Hub massless blades	15148 kg
Distance rotor to center tower axis	3.3 m
Nacelle mass without hub and blades	51170 kg
Nacelle mass center without rotor	0.145 m
Gear ratio	87.97

**Table 2:** Principal data of synchronous generator.

Synchronous generator model parameter	

Stator leakage reactance	0.06245 ohms
Quadrature axis 1 leakage reactance	0.05 ohms
Quadrature axis 2 leakage reactance	0.001 ohms
Main field leakage reactance	0.06245 ohms
Direct axis leakage reactance	0.001 ohms
Stator resistance	0.243 ohms
Quadrature axis 1 resistance	0.144 ohms
Quadrature axis 2 resistance	0.681 ohms
Main field resistance	0.75 ohms
Direct axis resistance	0.108 ohms
Stator quadrature axis reactance	0.8999 ohms
Stator direct axis reactance	1.03065 ohms
Base speed	60 *π* ohms
Number of poles	2
Moment of inertia	53.036 J s^2^
Excitation voltage	$\sqrt{2/3}$ ∗ 26000

**Table 3:** Comparison of the simulation results from the three control schemes.

		Gain scheduling PI control/LPV control in this research	LPV control in [8]/LPV control in this research

Power	Average produced power	0.94	1.006

Output load	Moment at blade root (*L*_2_-norm)	1.14	1.07
	Rotor torque (*L*_2_-norm)	1.04	1.03
	Pitch moment at the tower-top (*L*_2_and *L*_inf⁡_-norm)	1.38 and 1.38	0.80 and 0.99

Control input	Pitch angle (*L*_2_-norm)	1.13	1.005
	Generator torque (*L*_2_-norm)	1.05	1.06

## References

[1] Boukhezzar B., Siguerdidjane H. Robust multiobjective control of a variable speed wind turbine.

[2] Hand M. M., Balas M. J. (2002). Systematic controller design methodology for variable-speed wind turbines. *Technical Report*.

[3] Connor B., Leithead W. E., Grimble M. J. LQG control of a constant speed horizontal axis wind turbine.

[4] Munteanu I., Cutululis N. A., Bratcu A. I., Ceangǎ E. (2005). Optimization of variable speed wind power systems based on a LQG approach. *Control Engineering Practice*.

[5] Rocha R., Martins Filho L. S., Bortolus M. V. Optimal multivariable control for wind energy conversion system—a comparison between *H*
_2_ and *H*
_*∞*_ controllers.

[6] Leith D. J., Leithead W. E. Application of Nonlinear Control to a HAWT.

[7] Leith D. J., Leithead W. E. (1996). Appropriate realization of gain-scheduled controllers with application to wind turbine regulation. *International Journal of Control*.

[8] Østergaard K. Z., Stoustrup J., Brath P. (2009). Linear parameter varying control of wind turbines covering both partial load and full load conditions. *International Journal of Robust and Nonlinear Control*.

[9] Lescher F., Zhao J. Y., Martinez A. Multiobjective H_2_/H_∞_ control of a pitch regulated wind turbine for mechanical load reduction.

[10] Lescher F., Camblong H., Curea O., Briand R. LPV control of wind turbines for fatigue loads reduction using intelligent micro sensors.

[11] Shirazi F. A., Grigoriadis K. M., Viassolo D. (2012). Wind turbine integrated structural and LPV control design for improved closed-loop performance. *International Journal of Control*.

[12] Bobanac V., Jelavić M., Perić N. Linear parameter varying approach to wind turbine control.

[13] Inthamoussou F. A., Bianchi F. D., De Battista H., Mantz R. J. (2014). LPV wind turbine control with anti-windup features covering the complete wind speed range. *IEEE Transactions on Energy Conversion*.

[14] Meisami-Azad M., Grigoriadis K. M. (2013). Anti-windup linear parameter-varying control of pitch actuators in wind turbines. *Wind Energy*.

[15] Muhando E. B., Senjyu T., Uehara A., Funabashi T. (2011). Gain-scheduled *H*
_*∞*_ control for WECS via LMI techniques and parametrically dependent feedback part II: controller design and implementation. *IEEE Transactions on Industrial Electronics*.

[16] Qiao W., Yang X., Gong X. (2012). Wind speed and rotor position sensorless control for direct-drive PMG wind turbines. *IEEE Transactions on Industry Applications*.

[17] Corradini M. L., Ippoliti G., Orlando G. (2013). Robust control of variable-speed wind turbines based on an aerodynamic torque observer. *IEEE Transactions on Control Systems Technology*.

[18] Jonkman J. M., Buhl M. L. (2005). FAST user’s guide.

[19] Bianchi F. D., Battista H. D., Mantz R. J. (2006). *Wind Turbine Control Systems: Principles, Modeling and Gain Scheduling Design*.

[20] Laino D. J., Hansen A. C. (2002). *User’s Guide to the Wind Turbine Dynamics Computer Software AeroDyn*.

[21] Krause P. C., Wasynczuk O., Sudhoff S. D. (2002). *Analysis of Electric Machinery and Drive Systems*.

[22] Skogestad S., Postlethwaite I. (2005). *Multivariable Feedback Control Analysis and Design*.

[23] Gahinet P., Apkarian P., Chilali M. (1996). Affine parameter-dependent Lyapunov functions and real parametric uncertainty. *IEEE Transactions on Automatic Control*.

[24] Feron E., Apkarian P., Gahinet P. (1996). Analysis and synthesis of robust control systems via parameter-dependent Lyapunov functions. *IEEE Transactions on Automatic Control*.

[25] Apkarian P., Adams R. J. (1998). Advanced gain-scheduling techniques for uncertain systems. *IEEE Transactions on Control Systems Technology*.

[26] Simley E., Pao L. Y., Frehilich R., Jonkman B., Kelley N. Analysis of wind speed measurements using continuous wave LIDAR for wind turbine control.

[27] Dunne F., Simley E., Pao L. Y. (2011). LIDAR wind speed measurement analysis and feed-forward blade pitch control for load mitigation in wind turbines. *Subcontract Report*.

[28] MATLAB (2012). *Curve Fitting Toolbox User’s Guide*.

[29] MATLAB (1995). *LMI Control Toolbox for Use with MATLAB*.

[30] Jonkman J. M., Butterfield S., Musial W., Scott G. (2009). Definition of a 5-MW reference wind turbine for offshore system development.

[31] Hansen M. H., Hansen A., Larsen T. J., Øye S., Sørensen P., Fuglsang P. (2005). Control design for a pitch regulated variable speed wind turbine.

